# Creep modeling of composite materials based on improved gene expression programming

**DOI:** 10.1038/s41598-022-26548-6

**Published:** 2022-12-23

**Authors:** Hua Tan, Shilin Yan, Sirong Zhu, Pin Wen

**Affiliations:** 1 Department of Engineering Structure and Mechanics, School of Science, Wuhan University of Technology, Wuhan, 430070 China; 2 Hubei Key Laboratory of Theory and Application of Advanced Materials Mechanics, Wuhan University of Technology, Wuhan, 430070 China

**Keywords:** Evolutionary theory, Engineering, Materials science, Mathematics and computing, Physics

## Abstract

In this article, a new method for creep modeling and performance prediction of composite materials is presented. Since Findley power-law model is usually suitable for studying one-dimensional time-dependent creep of materials under low stress, an intelligent computing method is utilized to derive three temperature-related sub-functions, the creep model as a function of time and temperature is established. In order to accelerate convergence rate and improve solution accuracy, an improved gene expression programming (IGEP) algorithm is proposed by adopting the probability-based population initialization and semi-elite roulette selection strategy. Based on short-term creep data at seven temperatures, a bivariate creep model with certain physical significance is developed. At fixed temperature, the univariate creep model is acquired. R^2^, RMSE, MAE, RRSE statistical metrics are used to verify the validity of the developed model by comparison with viscoelastic models. Shift factor is solved by Arrhenius equation. The creep master curve is derived from time–temperature superposition model, and evaluated by Burgers, Findley and HKK models. R-square of IGEP model is above 0.98 that is better than classical models. Moreover, the model is utilized to predict creep values at t = 1000 h. Compared with experimental values, the relative errors are within 5.2%. The results show that the improved algorithm can establish effective models that accurately predict the long-term creep performance of composites.

## Introduction

Fiber reinforced polymer composites, as a class of widely used composite materials, have the advantages of high specific strength and modulus, fatigue and corrosion resistance, low density, light weight, which have been applied in the field of civil engineering, aerospace, automotive and construction industries, etc.^1,2^. In practical applications, they need to have a long service life. However, the viscoelastic properties of materials make the structures undergo creep behavior during long-term load-bearing, which affects the durability and reliability of composites. Creep is time-dependent deformation under constant stress. The mechanisms of creep deformation are different for each material but creep process may be generally described to include three stages: primary (transient), secondary (steady-state) and tertiary (accelerated) creep. In the primary stage, deformation increases rapidly and creep rate decreases over time. In the secondary stage, deformation is almost uniform and creep rate remains constant. In the tertiary stage, deformation and creep rate increases rapidly until the material ruptures after undergoing a total of strain within a period of time^3,4^. Therefore, the modeling research on creep performance has great theoretical significance.

At present, the models describing creep performance of composites can be divided into two categories: the first type is the physical model, it is based on creep mechanism of the material itself, and is established with the help of micro/meso-mechanics and thermodynamics, which mainly includes Maxwell model, Kelvin model, Burgers model, Boltzmann model, and Schapery model; the second type is the phenomenological model, it is a mathematical description of creep phenomenon, and is free from the constraint of fixed function forms and does not reflect the physical properties of creep, which mainly includes Findley model and time–temperature superposition model. Recently, there are more and more studies on these two types of models.

In physical model, Katouzian et al.^5^ used finite element method to simulate creep behavior of composite materials based on Schapery model. Rafiee and Mazhari^6^ developed Boltzmann model to obtain residual strength of pipes after 50 years for predicting long-term behavior of specific GFRP pipe subjected to internal pressure. Berardi et al.^7^ carried out creep experiments of fiber reinforced polymer laminates at room temperature, and established Burgers model of fibers. Jia et al.^8^ employed Burgers model and Weibull distribution function to analyze the effects of nano-fillers on creep and recovery properties of polypropylene/multi-walled carbon nanotube composites, and then long-term creep behavior of materials was predicted by time–temperature superposition model. Asyraf et al.^9^ found that Burgers model was very practical for explaining the elastic and viscoelastic behaviors of composite structures.

In phenomenological model, Zhang et al.^10^ employed four viscoelastic models to quantify the viscoelastic behavior of SCF/PEI composites, and then predict long-term creep behavior by time–temperature superposition model. Yang et al.^11^ evaluated long-term creep deformation and mechanical strength of tube by time–temperature superposition model and Findley model under expected service conditions over its entire lifetime. Harries et al.^12^ demonstrated a framework for evaluating creep behavior and buckling performance of GFRP, and obtained reliable Findley parameters. Ghosh et al.^13^ focused on the impact of multi-layer graphene reinforcement on mechanical performance of glass fiber/epoxy composites, and long-term creep performance at low temperature (30 °C) has been predicted by using accelerated deformation at elevated temperatures and time–temperature superposition model. Yu and Ma^14^ concentrated on the influence of loading rate and frequency/temperature on static flexural behavior and dynamic mechanical properties of injection molded GFPP, and the long-term durability of PP and GFPP was investigated by master curve of storage modulus constructed based on time–temperature superposition model. Asyraf et al.^15^ also discovered that Findley model was the most suitable for forecasting creep behaviours of wood and composite materials.

Most of creep models approximate time-dependent creep behaviour by a series of elastic spring and viscous dashpot elements that can be influenced by some factors such as temperature, stress, humidity and fiber morphology, which degrades the mechanical properties of composites. The low applicability of physical model and phenomenological model increases the difficulty of creep studies. Creep can be regarded as a complex evolution process with time. Therefore, gene expression programming developed by Ferreira^16^ is a genotype/phenotype evolutionary algorithm and attracts wide attention of scholars around the world. The individuals are encoded as linear strings of fixed length (genotype) that are afterwards expressed as nonlinear entities of different sizes and shapes (phenotype). It has rapidly become a powerful tool of automatic modeling without a large database or any predefined equations in the application of symbolic regression, time series prediction, data mining and many other fields^17^.

Recently, gene expression programming has been successfully applied to establish empirical models. For example, Murad^18,19^ applied gene expression programming to propose predictive model for shear strength of reinforced concrete columns subjected to biaxial cyclic loads. Moreover, Murad et al.^20^ introduced gene expression programming to develop simplified model for predicting flexural behavior of FRP reinforced concrete beams. They found that there was a good agreement between experimental results and numerical simulation. Babanajad et al.^21^ developed predictive models for true triaxial strength estimation of hardened concrete under general confinement configurations using gene expression programming. Iqbal et al.^22^ employed gene expression programming to develop empirical models for the prediction of mechanical properties of concrete with waste foundry sand. Wei and Xue^23^ proposed a new equation that could predict the permeability of tight carbonate rocks using gene expression programming. Hassani et al.^24^ presented fire resistance predictive model of steel-reinforced concrete composite columns by gene expression programming. Shahmansouri et al.^25^ studied gene expression programming to establish numerical models for compressive strength of GPC based on ground granulated blast-furnace slag, and validated the performance and predictability of proposed model by conducted sensitivity and parametric analysis. Mousavi et al.^26^ utilized gene expression programming to derive empirical model for the prediction of compressive strength of high performance concrete mixes. Mansouri et al.^27^ developed a framework for shear behavior of RC beam-column joints where a novel model was presented by gene expression programming. Beheshti Aval et al.^28^ estimated shear strength of short rectangular reinforced concrete columns using gene expression programming. Tarawneh et al.^29^ employed gene expression programming to establish accurate and reliable model to predict shear capacity of steel fiber-reinforced concrete beams. Kara^30^ presented an improved model to predict shear strength of FRP reinforced concrete beams without stirrups based on gene expression programming. Yeddula and Karthiyaini^31,32^ proposed a novel mathematical equation for predicting compressive strength of sialate/ferrosialate geopolymer mortars using gene expression programming. Güneyisi and Nour^33,34^ implemented gene expression programming to develop predictive model of axial capacity of concrete filled steel tube columns. Furthermore, some researchers utilized gene expression programming for predicting the strength of special concretes like lightweight concrete^35^, and recycled aggregate concrete^36^, etc. To the best of our knowledge, gene expression programming is very effective in the prediction of mechanical properties for solving many structural engineering problems^18–36^. There have been some studies involved in creep modeling based on classical viscoelastic models^5–15^. Therefore, the aim of this article is to simulate creep evolution process of composite materials to develop mathematical models by using gene expression programming. An intelligent evolutionary approach is employed instead of viscoelasticity-based approach. The physical model is generally used for theoretical analysis and has many limitations. The phenomenological model is difficult to reflect physical significance of creep and is relatively rigid. The low adaptability of these models leads to the proposal of intelligent computing methods. Gene expression programming has efficient nonlinear modeling capability without the guidance of prior knowledge. The novelty of the study consists of three aspects as algorithm improvement, model validation and performance prediction for providing design guidance.

Gene expression programming is improved from genetic algorithm and genetic programming. It contains all the genetic operators of traditional algorithm and introduces some new genetic operators that brings some challenges to the convergence rate and solution accuracy. When there are many terminal symbols in the head of gene, it is easy to generate invalid individuals; when the fitness function is selected, the lack of population diversity results in slow convergence, and it is easy to fall into local optimum. Therefore, an improved gene expression programming algorithm is developed. The probability-based population initialization is adopted to accelerate convergence rate, and the semi-elite roulette selection is utilized to improve solution accuracy. Furthermore, the creep tests are performed to obtain short-term experimental data, three temperature-related sub-functions of Findley model are derived from improved gene expression programming algorithm to establish bivariate creep model. Compared with classical viscoelastic models, the validity of univariate model is verified by four statistical metrics at fixed temperature. Lastly, creep master curve is drawn from time–temperature superposition model based on shift factor. The developed model is applied to predict long-term creep performance of composites so that high prediction accuracy of the model is validated.

## The proposed methodology

**Figure 1:**
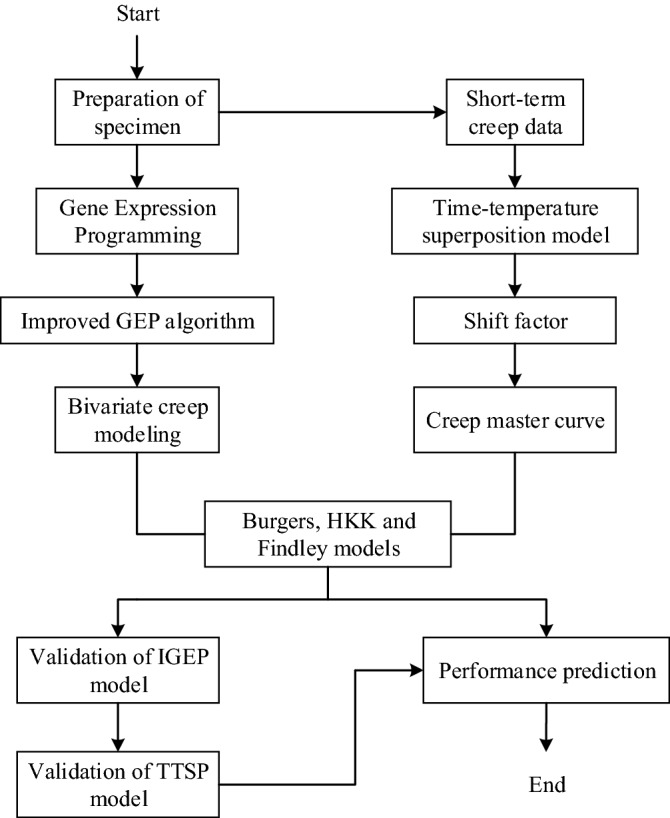
Flow chart of the research methodology.

In this section, the flow chart of overall research methodology is given as shown in Fig. 1. The methodology is divided into two stages: creep modeling and performance prediction. Further detail steps of the research are discussed in the subsequent subsections.

### Preparation of specimen

The matrix material of this experiment is m-benzene type unsaturated polyester resin FC518, which was supplied by Shanghai Fuchen Chemical Co., Ltd. The reinforcement materials are made up of alkali-free glass fibers, with the specifications of winding yarn 2400 Tex and chopped strand mat 450 g/m2, which were provided by Hebei Zhongyi Composite Materials Co., Ltd. The experimental specimens are: resin (R), fiber chopped strand mat (CSM) and fiber circumferential winding (FWC). According to the standard GB/T 1449–2005, INSTRON5828 is used to test the initial flexural strength (*σ*) of specimens. The resin mass content (*W*) of each specimen is tested based on the standard GB/T 2577–2005, and the results are given in Table 1. The size of each specimen is determined by the above-mentioned standard, the thickness *h* = 5 mm, the width *b* = (2.5 ± 0.5) *h*, and the length *L* = (18 ± 2) *h*. The constant load applied by INSTRON5848 universal testing machine is 20% of the initial flexural strength, and these testing data are automatically read by computer with a time interval of 0.1 s.

**Table 1:** Initial flexural strength and resin content of specimens.

Specimen	*σ*/MPa	*W*/%
R	90.9	100
CSM	165.8	70
FWC	933.0	28

### Overview of gene expression programming

**Figure 2:**
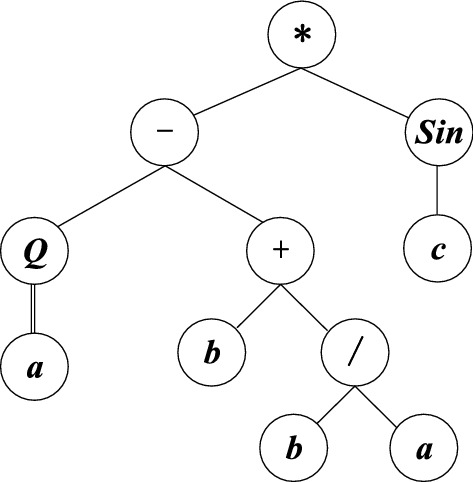
Expression tree corresponding to the genotype.

Gene Expression Programming (GEP) invented by Ferreira is derived and improved from genetic algorithm and genetic programming, it is an efficient tool for developing models and consists of chromosome with fixed length. Each gene in the chromosome contains a head $h$ and a tail $t$, there exists the following relationship:$t = h(n - 1) + 1$, $n$ is the total number of arguments within a function(maximum arity). The head of each gene contains both function symbols and terminal symbols (e.g.{+ , −, *, /,√,cos, tan, log, 6, *x*, *a*, b}). While the tail only contains terminal symbols that are made up of constants and variables (e.g.{8, *y*, *c*, d}). The chromosomes can be viewed as genomes that are modified through selection, crossover, mutation, transposition and recombination operations. GEP is developed based on two essential elements: chromosome and expression tree (ET). The genotype of GEP is chromosome, and the phenotype is ET that is composed of nonlinear entities with different sizes and shapes. For example, the chromosome consists of one gene, and the genotype of individual is: * − sinQ + cab/**bababbaaba**, the part in bold is the tail. The gene has a head length of 9 and a tail length of 10, so the total length of gene is 19. The genome and expression tree can be converted into each other in a certain way, as shown in Fig. 2.

$$\begin{aligned} & 0123456780123456789 \\ & {*} - {\text{sinQ + cab/}}{\mathbf{bababbaaba}} \\ & \left| { \leftarrow {\rm Head } \to } \right|\left| { \leftarrow {\rm Tail } \to } \right| \\ \end{aligned}$$

The mathematical equation corresponding to genotype can be expressed as:$\left( {\sqrt a { - }\left( {{\text{b}} + {b \mathord{\left/ {\vphantom {b a}} \right. \kern-0pt} a}} \right)} \right)*\left( {{\text{sinc}}} \right)$. Simultaneously, the fitness value $Fitness(i)$ of an individual $i$ is calculated, as given in the Eq. (1).

1 $$Fitness(i) = \sum\limits_{j = 1}^{n} {(M - \frac{{\left| {C(i,j) - T(j)} \right|}}{T(j)}*100)}$$

where $M$ is the selected range,$C(i,j)$ is the value returned by an individual $i$ for fitness case $j$(out of *n* fitness cases),$T(j)$ is the target value for fitness case $j$. If $C(i,j) = T(j)$, there is $Fitness(i) = n \cdot M$, the system can find the optimal model for itself by this way. Therefore. GEP greatly surpasses existing adaptive techniques^37^.

### 2.3 Proposition of improved GEP

**Figure 3:**
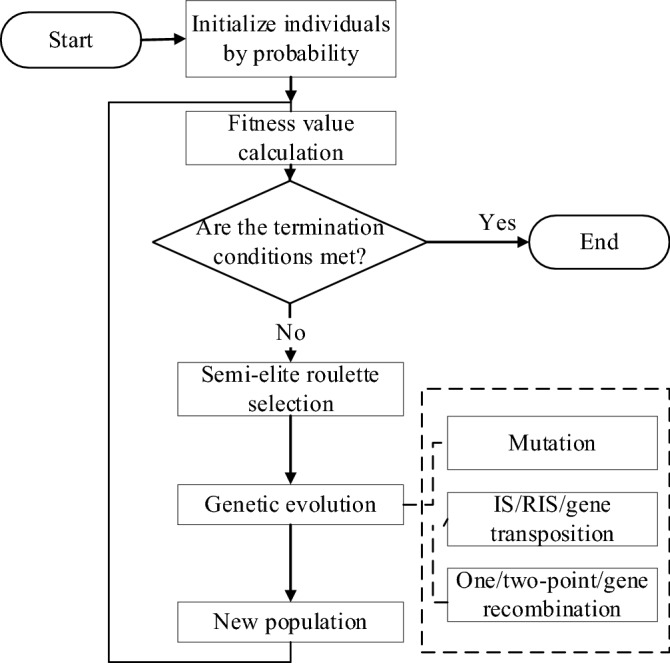
Flow chart of IGEP algorithm.

The individuals of GEP have linear genotype and non-linear phenotype. Simultaneously, GEP not only contains all the genetic operators of traditional evolutionary algorithm but also introduces some new operators, which brings some challenges to the convergence rate and solution accuracy. Although GEP algorithm has flexible encoding/decoding methods and evolutionary operations, when there are many terminal symbols in the head of gene, it is easy to generate invalid individuals; when the fitness function is selected, the lack of population diversity results in slow convergence, and it is easy to fall into local optimum. Therefore, this article proposes an improved GEP (IGEP) algorithm. The individuals are initialized by probability to accelerate convergence rate; the semi-elite roulette selection is performed to improve solution accuracy. Its flow chart is shown in Fig. 3.

The detailed steps of the algorithm are given in Table 2.

**Table 2:**
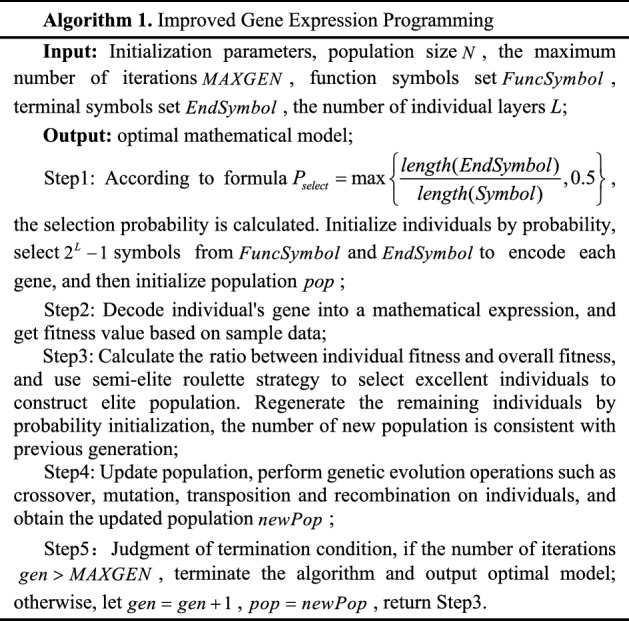
Description of IGEP algorithm.

### 2.3 Algorithm complexity analysis

The low time cost of IGEP algorithm is very important to be used to build the model. Given the maximum number of iterations is $MAXGEN$, the size of population is $N$, the size of elite population is $M$, the length of gene is $len$, and the size of sample data is $S$. As can be known from the algorithm, in Step 1, individuals with a length of $len$ are traversed and gene encoding is performed. Therefore, the time complexity of population initialization process is $O\left( {N \cdot len} \right)$. In Step 2, the fitness value of each individual is evaluated, so the time complexity is $O\left( {N \cdot S} \right)$. In Step3, firstly, the ratio of individual fitness to overall fitness is calculated, and its time complexity is $O\left( N \right)$; secondly, the semi-elite roulette strategy is utilized to select individuals, the time complexity is $O\left( {N^{2} } \right)$; thirdly, the sorting algorithm is employed to select elite population, and its time complexity is $O\left( {N\log \left( N \right)} \right)$; finally, the remaining individuals are regenerated with a time complexity of $O((N - M) \cdot len) \approx O(N \cdot len)$. Therefore, the total time complexity required in Step3 is $O\left( {\left( {N + \log \left( N \right) + len + 1} \right) \cdot N} \right)$. In Step 4, three genetic operations are all performed in parallel, when the genes are exchanged, its time complexity is $O\left( {N \cdot len} \right)$. In summary, the time complexity required for one iteration is $O(3N \cdot len + N \cdot S + N^{2} + N + N \cdot \log (N))$. After removing constant term and simplifying the formula, the total time complexity of all iterations is $O((len + S + N + \log (N)) \cdot N \cdot MAXGEN)$^38^.

### The physical model

#### Burgers model

**Figure 4:**
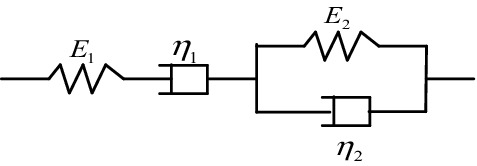
Schematic diagram of Burgers model.

Burgers model is a combination of Maxwell and Kelvin–Voigt elements, it is one of the most widely used models to give the relationship between morphology of composites and their creep behavior^39^, which is a four-element model, as shown in Fig. 4.

For the most general case of linear viscoelastic materials, the total creep strain is essentially the sum of three separate parts: $\varepsilon_{1}$ is the instantaneous elastic deformation; $\varepsilon_{2}$ is the delayed elastic deformation; $\varepsilon_{3}$ is the Newtonian flow, it is the same as the deformation of a viscous liquid that obeys Newton’s law of viscosity. The total strain $\varepsilon_{{\text{B}}} (t)$ as a function of time corresponds to the following Eq. (2). The creep constitutive equations of Burgers model take the basic forms:

2 $$\varepsilon_{{\text{B}}} (t) = \varepsilon_{1} + \varepsilon_{2} + \varepsilon_{3} = \frac{{\sigma_{0} }}{{E_{1} }} + \frac{{\sigma_{0} }}{{E_{2} }}\left( {1 - e^{{ - \frac{{E_{2} }}{{\eta_{2} }}t}} } \right) + \frac{{\sigma_{0} }}{{\eta_{1} }}t$$

3 $$C_{{\text{B}}} (t) = \frac{{\varepsilon_{{\text{B}}} \left( t \right)}}{{\sigma_{0} }} = \frac{1}{{E_{1} }} + \frac{1}{{E_{2} }}\left( {1 - e^{{ - \frac{{E_{2} }}{{\eta_{2} }}t}} } \right) + \frac{1}{{\eta_{1} }}t$$

where *t* denotes the time after loading, $\sigma_{0}$ is the applied stress, $C_{{\text{B}}} (t)$ is the creep compliance,$E_{i}$ and $\eta_{i}$ are the model parameters, $i{ = }1,2$.$E_{1}$ and $\eta_{1}$ are the elastic modulus and viscosity of Maxwell spring and dashpot, respectively; $E_{2}$ and $\eta_{2}$ are the elastic modulus and viscosity of Kelvin spring and dashpot, respectively.

#### HKK model

**Figure 5:**
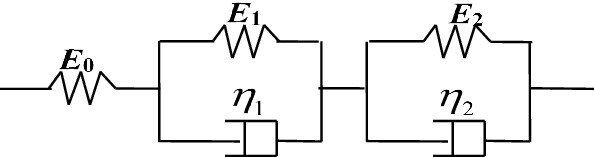
Schematic diagram of HKK model.

Various physical models are constructed through different combinations of elastic spring and viscous dashpot elements that can describe hysteresis and creep, such as Maxwell model and Kelvin model. HKK model is a combination of a Hooke spring body and two Kelvin models (called HKK), it describes creep process of composite materials, and its elements are shown in Fig. 5.

The constitutive equations of HKK model take the basic forms:

4 $$\varepsilon_{{\text{H}}} (t) = \frac{{\sigma_{0} }}{{E_{0} }} + \frac{{\sigma_{0} }}{{E_{1} }}\left( {1 - e^{{ - \frac{{E_{1} }}{{\eta_{1} }}t}} } \right) + \frac{{\sigma_{0} }}{{E_{2} }}\left( {1 - e^{{ - \frac{{E_{2} }}{{\eta_{2} }}t}} } \right)$$

5 $$C_{{\text{H}}} (t) = \frac{{\varepsilon_{{\text{H}}} \left( t \right)}}{{\sigma_{0} }} = \frac{1}{{E_{0} }} + \frac{1}{{E_{1} }}\left( {1 - e^{{ - \frac{{E_{1} }}{{\eta_{1} }}t}} } \right) + \frac{1}{{E_{2} }}\left( {1 - e^{{ - \frac{{E_{2} }}{{\eta_{2} }}t}} } \right)$$

where *t* is the time,$\sigma_{0}$ is the applied stress, $\varepsilon_{{\text{H}}} (t)$ is the total strain, $C_{{\text{H}}} (t)$ is the creep compliance, $E_{i}$ and $\eta_{j}$ are the model parameters, $i{ = }0,1,2$, $j{ = }1,2$.$E_{0}$ is the initial elastic modulus; $E_{1}$ and $E_{2}$ are the elastic moduli of Kelvin springs, respectively; $\eta_{1}$ and $\eta_{2}$ are the viscosities of Kelvin dashpots, respectively.

### The phenomenological model

#### Findley power-law model

The phenomenological model developed by Findley introduces a mathematical expression to describe creep behavior of composite materials that is more suitable for the prediction of creep deformation, it can effectively predict mechanical performance of composites. In this model, the creep response can be divided into time-independent and time-dependent strains, creep strain can be expressed as follows:

6 $$\varepsilon \left( t \right) = \varepsilon_{0} + \varepsilon_{c} t^{n} \Rightarrow C\left( t \right) = C_{0} + mt^{n}$$

where $\varepsilon_{0}$ is the initial stress-dependent and time-independent elastic strain,$\varepsilon_{c}$ is a coefficient related to stress and temperature, *t* is the time, *n* is a stress-independent and temperature-dependent material constant^40^. Under constant stress, the subsequent form (7) could be derived, where *C*_0_ is the initial temperature-dependent creep, *m* is a temperature-related coefficient, and *n* is a dimensionless material parameter that is dependent of temperature. Since the specific mathematical form of Findley model with time and temperature has not been deduced in the theoretical analysis, at different temperatures, *C* can be determined as a bivariate function of both time and temperature. Therefore, Findley model is considered as a modeling framework, the modified model is represented as:

7 $$C(T,t) = C_{0} (T) + m(T)t^{n(T)}$$

#### Time-temperature superposition model

Assuming that creep compliance is a function related to time and temperature, the creep behavior of composites at low temperature for a long time can be predicted by using short-term creep data at high temperatures. The creep compliance curve $C\left( {T_{ref} ,t/\phi_{T} } \right)$ at reference temperature $T_{ref}$ can be constructed by shifting short-term compliance curve $C\left( {T_{i} ,t} \right)$ at different temperatures along the logarithmic time axis by shift factor $\phi_{T}$, and so the smooth creep master curve is derived, which is time-temperature superposition (TTSP), the calculation equation is as follows:

8 $$C\left( {T_{i} ,t} \right) = C\left( {T_{ref} ,t/\phi_{T} } \right)$$

where $C\left( {T_{i} ,t} \right)$ is the creep compliance,$T_{i}$ is different testing temperatures, *t* is the time,$T_{ref}$ is reference temperature,$\phi_{T}$ is shift factor.

Assuming the activation energy is constant, time-temperature shift factor $\phi_{T}$ is obtained to construct creep master curve, it is in good quantitative agreement with the Arrhenius equation, the formula is given in (9), which provides a reliable method for predicting long-term creep performance of composite materials.

9 $$\lg \phi_{T} = \frac{{E_{a} }}{R}\left( {\frac{1}{T} - \frac{1}{{T_{ref} }}} \right)\lg \left( e \right)$$

where $E_{a}$ is activation energy [${\text{kJmol}}^{{ - 1}}$], *R* is the universal gas constant with a value of $8.314 \times 10^{ - 3} {\text{ kJK}}^{{ - 1}} {\text{mol}}^{{ - 1}}$,$T$ is the testing temperature [K]. Equation (9) is applicable for temperature below glass transition temperature.

## Creep data and experimental settings

### Data description

**Figure 6:**
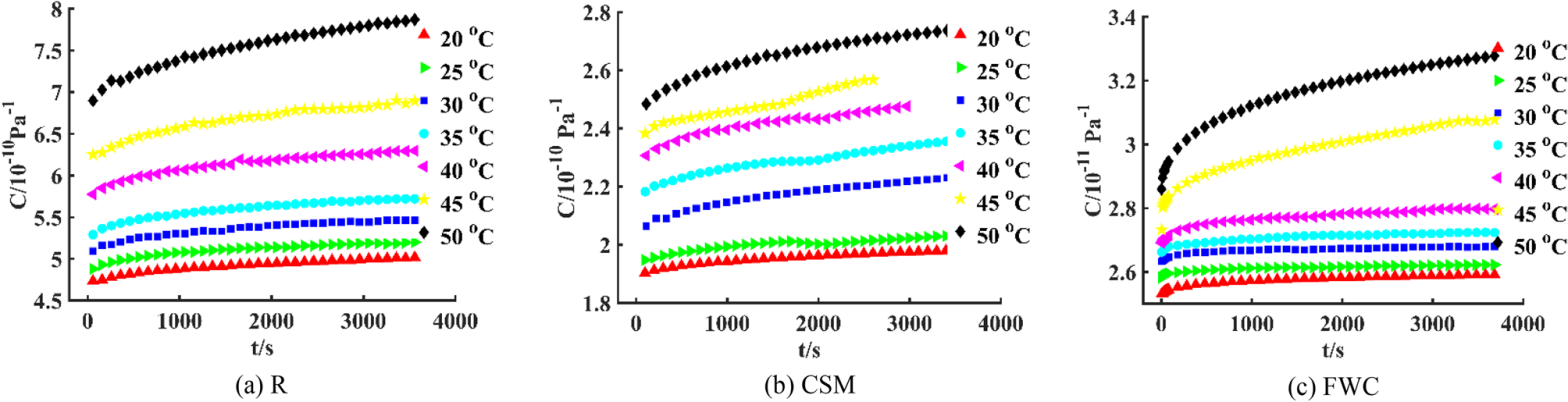
Creep compliance *C*-time *t* curves of three specimens at seven temperatures.

Three-point bending tests are carried out under constant load. The temperatures of R, CSM and FWC specimens are set to 20 °C, 25 °C, 30 °C, 35 °C, 40 °C, 45 °C, and 50 °C, respectively. According to the standards, these specimens need to be maintained within a constant temperature chamber for 20 min before testing to ensure that the experimental temperature is reached. Short-term (1 h) flexural creep performances of three specimens are tested at seven temperatures, and creep data ranging from 0 to 3600 s are obtained. The resin content of R specimen is 100%, without any constraint of reinforcement materials, so the creep compliance and creep growth rate are the largest, and its creep resistance is the weakest; the resin content of FWC specimen is the lowest, and its continuous fibers have the strongest constraint effect on resin deformation, so its creep compliance is the smallest and creep resistance is the strongest; the resin content of CSM specimen is relatively high, many interfaces lead to stress concentration, and the constraint effect of chopped fibers on resin is not as strong as that of continuous fibers, so its creep compliance and creep resistance are between the two. Therefore, the creep compliance *C*-time *t* curves of R, CSM and FWC specimens could be drawn, as shown in Fig. 6.

### Experimental settings

Various parameters are involved in the establishment of IGEP model, and affect generalization capability of the model. In order to get a more accurate IGEP model and reduce the time complexity, appropriate parameters need to be set for problem solving, including fitness function, the number of iterations, population size, the number of genes, linking function and probabilities of genetic operators. Based on multiple trials, the final parameters selected for IGEP algorithm are given in Table 3.

**Table 3:** The parameter settings for IGEP algorithm.

Parameters	Setting values
Fitness function	*R*^2^, *RMSE*
Max number of iterations	200,000
Population size	30
Function set	$\{ + , - ,*,/,e^{x} ,1/x, - x,x^{2} ,x^{3} ,x^{4} \}$
Terminal set	$t,T,c_{1} ,c_{2} ,c_{3} ,c_{4} ,c_{5}$
Chromosome	*h* = 7, *t* = 8, number of genes = 3
Linking function	Addition
One-point recombination	0.00277
Two-point recombination	0.00277
Gene recombination	0.00277
IS transposition	0.00546
RIS transposition	0.00546
Gene transposition	0.00546
Mutation rate	0.00138

### Evaluation metric

Four evaluation metrics, namely, coefficient of determination R-squared *R*^2^, root mean square error *RMSE*, mean absolute error *MAE* and relative square root error *RRSE*, are used to evaluate the performance and compare prediction accuracy of models. These criteria are calculated as follows:

9 $$R^{2} = \frac{{\sum\limits_{i = 1}^{n} {\left( {\hat{y}_{i} - \overline{y}} \right)^{2} } }}{{\sum\limits_{i = 1}^{n} {\left( {y_{i} - \overline{y}} \right)^{2} } }},\;RMSE = \sqrt {\frac{1}{n}\sum\limits_{i = 1}^{n} {\left( {\hat{y}_{i} - y_{i} } \right)^{2} } }$$

10 $$MAE = \frac{1}{n}\sum\limits_{i = 1}^{n} {\left| {\hat{y}_{i} - y_{i} } \right|} ,RRSE = \sqrt {\frac{{\sum\limits_{i = 1}^{n} {\left( {\hat{y}_{i} - y_{i} } \right)^{2} } }}{{\sum\limits_{i = 1}^{n} {\left( {\hat{y}_{i} - \overline{y}} \right)^{2} } }}}$$

where *n* is the number of data points, $y_{i}$ is the measured value, $\overline{y}$ is the average value, and $\hat{y}_{i}$ is the predicted value. *R*^2^ measures the degree of correlation, the larger the value of *R*^2^, the better the performance of model; *RMSE* is a measure of the residual variance, lower *RMSE* represents more accurate estimation; the smaller the values of *MAE* and *RRSE*, the better the performance of model.

## Creep modeling results and validation of the model

The experiments are implemented on a PC with Intel Core i5-4460 3.20 GHz CPU, 8 GB memory, Win7 64-bit operating system, and the software environment is MATLAB R2016a.

### Time-temperature bivariate creep modeling results

Based on short-term creep data from 0 to 3600 s, Findley model is modified to be expressed as a function of time and temperature by IGEP algorithm. Therefore, the time–temperature bivariate IGEP models for three specimens are established, the modeling results are given in Table 4, where $a_{i}$(i = 1, 2, 3,…) is the model parameter, *C*_0_, *m* and *n* are three sub-functions related to temperature *T* respectively, and *R*^2^ of three models are above 0.98. Moreover, these modified Findley equations are suitable for describing the creep behavior in all isothermal conditions, although the kernel function is different at each temperature. It can be known that at fixed temperature, when the time tends to infinity, IGEP model of specimens is provided with the physical properties of creep. In addition, the first-order and second-order derivative values approach zero, IGEP model satisfies the variation law that creep strain increases monotonically and tends to be stable.

**Table 4:** IGEP creep models for three specimens.

Specimen	IGEP model	*R*^2^	*RMSE*	*MAE*	*RRSE*
R	$C{(}T{,}t{) = }T{ + }a_{1} { + (}T{ + }a_{2} {)}t^{{a_{3} e^{T} {/(}e^{{a_{4} {/}T}} { - }T{ + }a_{5} {)}}}$	0.9928	0.0487	0.0430	0.0848
CSM	$C{(}T{,}t{) = }a_{1} + T/(T + 1){ + (}e^{{e^{{e^{{e^{{(a_{2} + a_{3} T)}} }} }} }} + T{)}t^{{a_{4} T}}$	0.9962	0.0148	0.0109	0.0617
FWC	$C{(}T{,}t{) = (}T{ + }a_{1} {)/}a_{2} { + }e^{{e^{{T/a_{3} }} }} t^{{e^{T} {/}a_{4} }}$	0.9867	0.0264	0.0172	0.1154

**Figure 7:**
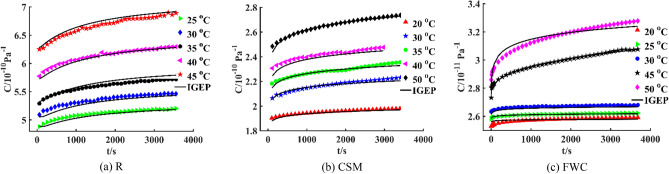
Fitting curves for three specimens.

**Figure 8:**
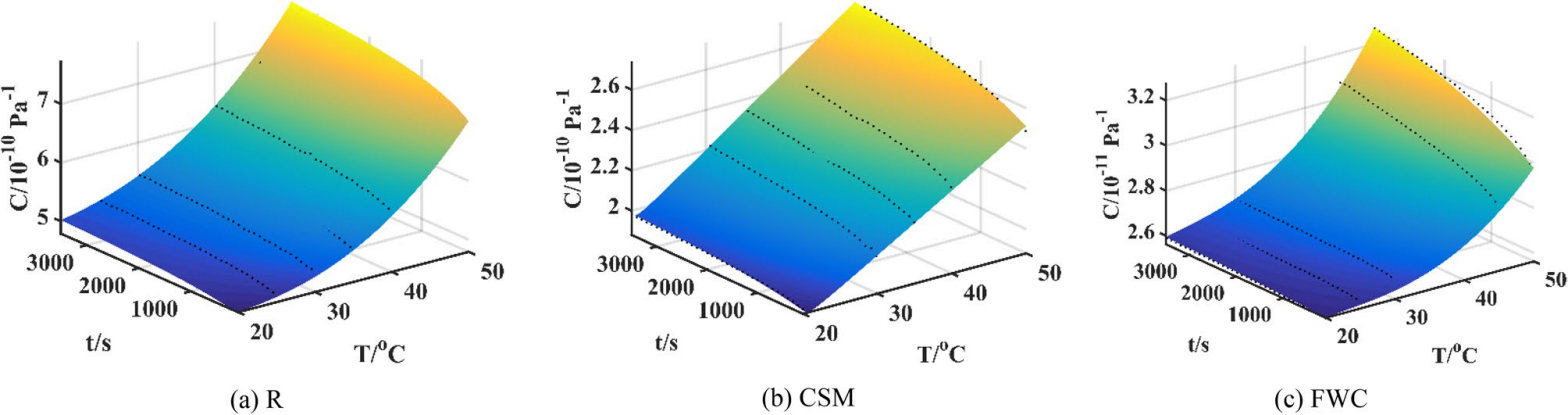
Fitting surfaces for three specimens.

R specimen is analyzed, creep compliance values at 25 °C, 30 °C, 35 °C, 40 °C and 45 °C are used as training dataset, and a time-temperature bivariate creep model is established. The fitting curve and fitting surface are plotted in Figs. 7a and 8a. The coefficient of determination *R*^2^ is 0.9928 obtained by IGEP model, the values of *RMSE*, *MAE* and *RRSE* are 0.0487, 0.0430 and 0.0848 for training phase, respectively. Moreover, creep compliance values at 20 °C and 50 °C are used as validation dataset, the coefficient of determination *R*^2^ is 0.9983 obtained by IGEP model, the values of *RMSE*, *MAE* and *RRSE* are 0.0538, 0.0397 and 0.0407 for validation phase, respectively, as provided in Table 5. The statistical metric values are effectively similar for training and validation set, the results indicate high generalization capacity and precise prediction ability of IGEP model. It can be found that there is a good coincidence between experimental data and fitting curves with low errors.

**Table 5:** Metric values of training and validation data for R specimen.

Data	*R*^*2*^	*RMSE*	*MAE*	*RRSE*
25 °C	0.7993	0.0355	0.0315	0.4480
30 °C	0.6880	0.0543	0.0510	0.5586
35 °C	0.7533	0.0543	0.0497	0.4967
40 °C	0.9295	0.0357	0.0267	0.2655
45 °C	0.8880	0.0585	0.0563	0.3347
Training	0.9928	0.0487	0.0430	0.0848
20 °C	0.8791	0.0263	0.0229	0.3477
50 °C	0.9208	0.0714	0.0564	0.2814
Validation	0.9983	0.0538	0.0397	0.0407

Similarly, CSM specimen is analyzed, creep compliance values at 20 °C, 30 °C, 35 °C, 40 °C and 50 °C are used as training dataset, and a bivariate creep model is established. The fitting curve and fitting surface are plotted in Figs. 7b and 8b. The coefficient of determination *R*^2^ is 0.9962 obtained by IGEP model, the values of *RMSE*, *MAE* and *RRSE* are 0.0148, 0.0109 and 0.0617 for training phase, respectively. Moreover, creep compliance values at 25 °C and 45 °C are used as validation dataset, *R*^2^ is 0.9638 obtained by IGEP model, the values of *RMSE*, *MAE* and *RRSE* are 0.0458, 0.0421 and 0.1903 for validation phase, respectively, as provided in Table 6. Simultaneously, FWC specimen is analyzed, creep compliance values at 20 °C, 25 °C, 30 °C, 45 °C and 50 °C are used as training dataset, and a bivariate creep model is established. The fitting curve and fitting surface are plotted in Figs. 7c and 8c. The coefficient of determination *R*^2^ is 0.9867 obtained by IGEP model, the values of *RMSE*, *MAE* and *RRSE* are 0.0264, 0.0172 and 0.1154 for training phase, respectively. Moreover, creep compliance values at 35 °C and 40 °C are used as validation dataset, *R*^2^ is 0.9242 obtained by IGEP model, the values of *RMSE*, *MAE* and *RRSE* are 0.0109, 0.0089 and 0.2753 for validation phase, respectively, as provided in Table 7. The high *R*^2^ and low *RMSE*, *MAE* and *RRSE* values demonstrate that the developed IGEP models are trained effectively and can well describe creep performance of composites at different temperatures.

**Table 6:** Metric values of training and validation data for CSM specimen.

Data	*R*^2^	*RMSE*	*MAE*	*RRSE*
20 °C	0.8215	0.0085	0.0078	0.4225
30 °C	0.9264	0.0120	0.0099	0.2713
35 °C	0.9177	0.0128	0.0088	0.2868
40 °C	0.6416	0.0262	0.0241	0.5987
50 °C	0.9766	0.0104	0.0059	0.1529
Training	0.9962	0.0148	0.0109	0.0617
25 °C	0.6043	0.0190	0.0178	0.5957
45 °C	0.6975	0.0281	0.0245	0.5500
Validation	0.9638	0.0458	0.0421	0.1903

**Table 7:** Metric values of training and validation data for FWC specimen.

Data	*R*^2^	*RMSE*	*MAE*	*RRSE*
20 °C	0.7251	0.0098	0.0093	0.5243
25 °C	0.9136	0.0034	0.0029	0.2939
30 °C	0.7747	0.0089	0.0070	0.4746
45 °C	0.7359	0.0474	0.0367	0.5139
50 °C	0.9430	0.0299	0.0242	0.2387
Training	0.9867	0.0264	0.0172	0.1154
35 °C	0.5837	0.0120	0.0097	0.6452
40 °C	0.9107	0.0098	0.0081	0.2988
Validation	0.9242	0.0109	0.0089	0.2753

### Validation of IGEP model

**Figure 9:**
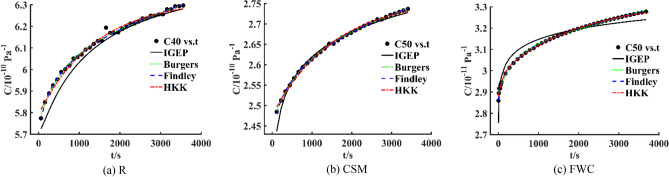
Creep models for R, CSM and FWC specimens at fixed temperature 40 °C, 50 °C and 50 °C.

Due to low adaptability of classical models under complex conditions, the previous research on creep performance is mostly univariate creep model related to time or creep master curve drawn from TTSP. Therefore, IGEP algorithm is utilized to establish a time-temperature bivariate model and get the fitting surface. When a certain temperature is fixed, the bivariate creep model is analyzed by dimension reduction. Then three-dimensional surface is converted into two-dimensional curve, the univariate model as a function of time is acquired. To further verify the validity of bivariate creep model, IGEP model for R specimen is analyzed, the creep curve at fixed temperature 40 °C is obtained. Compared with Burgers model, Findley model and HKK model, the curve fitting results are plotted in Fig. 9a. At the same time, four metric values of *R*^2^, *RMSE*, *MAE* and *RRSE* are calculated, as given in Table 8. Similarly, IGEP models for CSM and FWC specimens are analyzed by dimension reduction, and the creep curves at 50 °C are obtained. Compared with viscoelastic models, the curve fitting results are plotted in Fig. 9b,c. Simultaneously, the overall performance of IGEP model can be validated by four metrics *R*^2^, *RMSE*, *MAE* and *RRSE*, the values are provided in Table 8.

**Table 8:** Evaluation Metric values of creep models for R, CSM and FWC specimens at 40 °C, 50 °C and 50 °C, respectively.

Temperature	model	*R*^2^	*RMSE*	*MAE*	*RRSE*
R_40 °C	IGEP	0.9295	0.0357	0.0267	0.2655
	Burgers	0.9953	0.0092	0.0063	0.0688
	Findley	0.9957	0.0088	0.0055	0.0658
	HKK	0.9900	0.0135	0.0104	0.1002
CSM_50 °C	IGEP	0.9766	0.0104	0.0059	0.1529
	Burgers	0.9994	0.0016	0.0014	0.0239
	Findley	0.9993	0.0018	0.0014	0.0270
	HKK	0.9966	0.0040	0.0033	0.0585
FWC_50 °C	IGEP	0.9430	0.0299	0.0242	0.2387
	Burgers	0.9962	0.0077	0.0052	0.0614
	Findley	0.9995	0.0028	0.0019	0.0226
	HKK	0.9986	0.0047	0.0031	0.0372

Since most of creep models are time-related univariate models, and there are few models with multiple variables, a new bivariate modeling program is developed by IGEP in this work, the effect of temperature is introduced into the traditional Findley power-law creep equation. It can be clearly seen from the table that *R*^2^ values of univariate IGEP model for three specimens are above 0.92 by dimension reduction analysis, the coefficient of determination of four models are relatively high and close to each other. The results show that the fitting curve of IGEP model is almost in good agreement with experimental data.

### Time-temperature superposition creep modeling results

Calculating activation energy is a very useful technique to estimate shift factor for time-temperature superposition without constructing complete master curve. The activation energies $E_{a}$ of R, CSM and FWC specimens are obtained by dynamic mechanical thermal analysis to be 365.50 kJ/mol, 337.07 kJ/mol and 319.66 kJ/mol, respectively. Assuming $E_{a}$ is valid only below material's glass transition temperature. In this article, 23 °C is selected as reference temperature $T_{ref}$. Since some experimental temperatures are higher than reference temperature 23 °C, others are lower than 23 °C. For $T > T_{ref}$, the logarithm of shift factor $\lg \phi_{T}$ is negative resulting in right-shifted creep compliance curve. On the contrary, for $T < T_{ref}$, the logarithm of shift factor $\lg \phi_{T}$ is positive resulting in left-shifted creep curve. According to Arrhenius equation, the logarithm of shift factor for three specimens are calculated as given in Table 9. It is clearly seen that the order of $\lg \phi_{T}$ for three specimens at the same temperature is as follows: $\left| {{\text{lg}}\left( {\text{R}} \right)} \right| > \left| {{\text{lg}}\left( {{\text{CSM}}} \right)} \right| > \left| {{\text{lg}}\left( {{\text{FWC}}} \right)} \right|$. The larger the logarithm of shift factor, the greater the effect of temperatures on creep performance of composites. Therefore, the sensitivity of creep to temperatures for three specimens is: R > CSM > FWC.

**Table 9:** Logarithm of shift factor for three specimens at seven temperatures.

Parameter	20 °C	25 °C	30 °C	35 °C	40 °C	45 °C	50 °C
$\lg \phi_{T}$ (R)	1.5191	− 0.9958	− 3.4277	− 5.7808	− 8.0586	− 10.2649	− 12.4030
$\lg \phi_{T}$ (CSM)	1.4010	− 0.9183	− 3.1611	− 5.3311	− 7.4318	− 9.4665	− 11.4382
$\lg \phi_{T}$ (FWC)	1.3286	− 0.8709	− 2.9978	− 5.0558	− 7.0480	− 8.9775	− 10.8474

**Figure 10:**
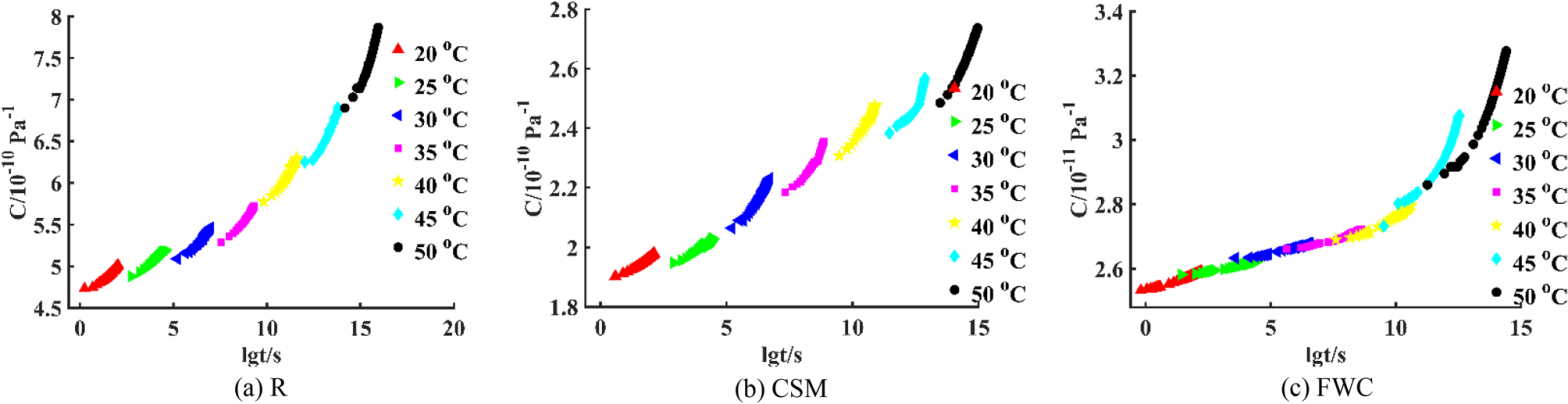
Creep master curves for three specimens.

**Figure 11:**
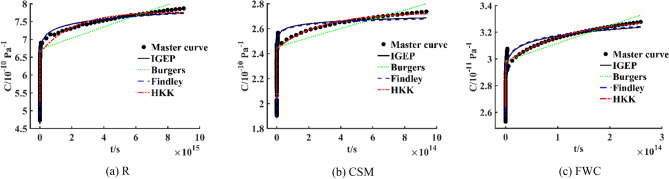
Creep master curves and fitting curves for three specimens.

When short-term experimental data of creep compliance *C*-time *t* at seven temperatures are used, creep master curve of R, CSM and FWC specimens can be derived from TTSP, as shown in Fig. 10. Findley model is a parametric phenomenological model suitable for creep behaviour under low stress conditions. Burgers model and HKK model are classical physical models. At present, there are different methods for master curve fitting. The abscissa axis in Fig. 10 represents the logarithm of time $\lg t$. In order to facilitate the observation, the abscissa axis is converted into time *t*, and is plotted to provide reference for engineering structural design. IGEP model and viscoelastic models are established by fitting the data on creep master curve for three specimens. The results are shown in Fig. 11. Moreover, the metric values of four models are calculated, as given in Table 10.

**Table 10:** Evaluation metric values of creep models for three specimens at reference temperature 23 °C based on TTSP.

Specimen	model	*R*^2^	*RMSE*	*MAE*	*RRSE*
R	IGEP	0.9950	0.0621	0.0486	0.0705
	Burgers	0.9245	0.2420	0.2060	0.2748
	Findley	0.9943	0.0666	0.0546	0.0756
	HKK	0.9328	0.2283	0.1885	0.2592
CSM	IGEP	0.9818	0.0328	0.0261	0.1350
	Burgers	0.9042	0.0752	0.0616	0.3096
	Findley	0.9787	0.0355	0.0307	0.1460
	HKK	0.9096	0.0730	0.0566	0.3007
FWC	IGEP	0.9835	0.0252	0.0159	0.1286
	Burgers	0.9311	0.0514	0.0446	0.2625
	Findley	0.9803	0.0275	0.0200	0.1402
	HKK	0.9345	0.0501	0.0418	0.2559

Apparently, it can be revealed that *R*^2^ of IGEP model is above 0.98, and the curve fits well with experimental data. *R*^2^, *RMSE*, *MAE* and *RRSE* are used as evaluation metrics, the fitting effect of IGEP model is better than that of Findley model, and far better than that of Burgers model and HKK model, indicating that IGEP model can well describe long-term creep performance of composite materials. When the creep compliance values of R and CSM specimens are enlarged by 10^10^ times, and creep compliance values of FWC specimen are enlarged by 10^11^ times, the model parameters are figured out with the help of computational software Origin 2018, the results are provided in Tables 11, 12 and 13.

**Table 11:** Burgers model parameters for creep master curve.

Specimen	$E_{1}$/(GPa)	$E_{2}$/(GPa)	$\eta_{1}$/(GPa*s)	$\eta_{2}$/(GPa*s)	$R^{2}$
R	1.902	6.829	63.950E15	13.896E11	0.9245
CSM	4.897	24.345	26.945E15	89.337E8	0.9042
FWC	37.83	292.77	755.63E14	147.40E11	0.9311

**Table 12:** HKK model parameters for creep master curve.

Specimen	$E_{0}$/(GPa)	$E_{1}$/(GPa)	$E_{2}$/(GPa)	$\eta_{1}$/(GPa*s)	$\eta_{2}$/(GPa*s)	$R^{2}$
R	1.903	7.342	8.621	12.150E11	17.808E15	0.9328
CSM	4.898	34.725	25.113	94.510E14	85.046E8	0.9096
FWC	37.83	304.79	298.69	141.99E11	356.93E14	0.9345

**Table 13:** Findley model parameters for creep master curve.

Specimen	$C_{0}$	$m$	$n$	$R^{2}$
R	4.605	0.2543	0.0683	0.9943
CSM	0.5153	1.3420	0.0138	0.9787
FWC	2.554	0.0208	0.1056	0.9803

### Validation of TTSP model and performance prediction

Since creep experiments at room temperature need to take a long time, the accelerated characterization of long-term creep behavior is performed. Shift factor is solved by Arrhenius equation, the short-term creep data at high temperatures could be used to predict long-term creep performance at low temperature. In order to verify the validity of TTSP model, under constant load, the long-term creep tests on 0–1000 h at reference temperature 23 °C are carried out, and the corresponding creep experimental data for R, FWC and CSM specimens are measured to compare with creep master curve obtained based on TTSP.

When the creep compliance at *t* = 1000 h is selected for analysis, four models are established by fitting master curve to predict the values at *t* = 1000 h. The predicted value of TTSP model is compared with experimental value at 23 °C, and then the relative error δ_TTSP_ is calculated, the results are provided in Tables 14 and 15. It can be seen that relative error δ_TTSP_ predicted by TTSP model for R specimen is 5.18%; relative error δ_TTSP_ for CSM specimen is 2.22%; and relative error δ_TTSP_ for FWC specimen is 1.15%, all are within 6%. It is well proven that long-term flexural creep life of composites can be accurately predicted through an accelerated testing method at high temperatures.

**Table 14:** The comparison between predicted value and experimental value at *t* = 1000 h.

Specimen	*C*_tested_	*C*_TTSP_	*C*_IGEP_	*C*_Burgers_	*C*_Findley_	*C*_HKK_
R	5.6203	5.3292	5.3329	5.2579	5.3182	5.2549
CSM	2.1559	2.2037	2.1690	2.0459	2.1693	2.0457
FWC	2.6493	2.6797	2.6631	2.6436	2.6563	2.6434

**Table 15:** Relative error δ predicted by creep models at *t* = 1000 h.

Specimen	*δ*_TTSP_ (%)	*δ*_IGEP_ (%)	*δ*_Burgers_ (%)	*δ*_Findley_ (%)	*δ*_HKK_ (%)
R	5.18	5.11	6.45	5.38	6.50
CSM	2.22	0.61	5.10	0.62	5.11
FWC	1.15	0.52	0.21	0.26	0.22

However, it is clearly seen from Table 14 that the prediction effect of IGEP model for R specimen is better than that of TTSP model and Findley model, far better than that of Burgers model and HKK model; the prediction effect of IGEP model for CSM specimen is comparable to that of Findley model, better than that of TTSP model, and far better than that of Burgers model and HKK model; the prediction effect of IGEP model for FWC specimen is better than that of TTSP model, and comparable to that of other creep models. It is concluded that IGEP model is a better way to simulate creep master curve.

At the same time, taking relative error δ between creep value predicted by each model and experimental value at *t* = 1000 h as a statistical metric, it can be seen from Table 15 that the relative error δ_IGEP_ of IGEP model for R specimen is the smallest, it is 5.11%; the relative error δ_IGEP_ of IGEP model for CSM specimen is almost the same as the error δ_Findley_ of Findley model, it is 0.61%; at *t* = 1000 h, the prediction effect of each model for FWC specimen is better than that of TTSP model, and the relative error δ is very small, all are below 0.6%. The predicted values are extremely consistent with experimental values. The experiments and theory are integrated to verify the validity of accelerated characterization method. The comparison of developed models and accelerated testing results indicates that IGEP model has better prediction accuracy than Burgers, Findley and HKK models in describing long-term creep performance of composite materials.

### Discussion

Creep modeling of composite materials is a subject widely studied in the field of material science and engineering. This article only investigates the effect of time and temperature on flexural creep behavior of composites that is very important to the service life. However, under the complex conditions, there are many factors involved in creep failure of materials, such as humidity, atomic migration and diffusion, crack initiation and propagation, fiber morphology and orientation, there exists uncertainty in creep properties of composites, so that the empirical prediction model is not accurate enough. In addition, the joint effect of various factors makes it difficult to simulate the evolution process of creep from a microscopic perspective. Therefore, a swarm intelligent algorithm can be utilized to establish mathematical relationship model between multiple factors and output from a macroscopic perspective. The randomness and fuzziness of creep are not considered that results in the failure of classical models. So the fuzzy random method is used to improve the traditional particle swarm algorithm to obtain an efficient model to describe creep performance^41^. The operation of instrument and the test of specimen result in certain errors in the data obtained. The creep test under constant load is performed, but the load is variable in practical application. An effective model for describing creep properties of composites under step loading and unloading conditions is established to provide theoretical support for deformation analysis and long-term stability^42^.

The intelligent evolutionary algorithm is easy to implement and has strong scalability by selecting different basis functions such as exponential function and power function. When there are few experimental samples, the useful information can still be analyzed and extracted from the data, so that the testing workload in the process of creep modeling is reduced. The modeling of alternative methods proves that the prediction of machine learning algorithm is superior to other methods in the literature^43^, it has wider engineering applicability and higher prediction accuracy in describing long-term creep performance of composites. GEP is an efficient evolutionary algorithm, it can be regarded as a promising approach to devise empirical models based on experimental phenomena and variation laws, Since the creep experiments at room temperature need to take a long time, the application of accelerated characterization method can reduce its time cost by short-term creep data. Although mechanical testing is one of the most direct ways to study mechanical properties of materials, the time-consuming and sophisticated creep tests could be avoided through computer simulation using GEP.

## Conclusions

To summarize this article, an intelligent computing method is proposed for creep modeling of composite materials. In order to derive three temperature-related sub-functions of Findley model, an improved GEP algorithm is developed to establish bivariate model. The probability-based population initialization and semi-elite roulette selection are adopted to accelerate convergence rate and improve solution accuracy. Moreover, compared with Burgers, Findley and HKK models, the validity of univariate model at fixed temperature is verified by *R*^2^, *RMSE*, *MAE* and *RRSE* metrics. Lastly, the short-term creep curves are plotted as creep master curve based on shift factor, the relative error at *t* = 1000 h is used as a statistical metric. IGEP model established by fitting master curve has lower prediction errors for three specimens, all are within 6%. The experimental results indicate that IGEP model can accurately predict long-term creep performance of composite materials. This work not only expands the application field of GEP algorithm, but also provides a new method for creep modeling.

In future work, except for TTSP, other superposition models could be extended and are reasonably studied to accelerate the characterization of long-term performance. When the effect of fiber content and surface treatment on creep properties of composites is studied further, GEP algorithm would be efficiently utilized to develop multivariable creep model as a function of temperature, stress and fiber, which is of great significance to investigate creep behavior for the design and life prediction.

## Data Availability

The data used to support the findings of this study are available from the corresponding author upon reasonable request.

## References

[1] D'Ambrisi A, Mezzi M, Feo L, Berardi VP (2014). Analysis of masonry structures strengthened with polymeric net reinforced cementitious matrix materials. Compos. Struct..

[2] Perrella M, Berardi VP, Cricrì G (2018). A novel methodology for shear cohesive law identification of bonded reinforcements. Compos. Part. B-Eng..

[3] Bouziadi F, Boulekbache B, Haddi A, Hamrat M, Djelal C (2019). Finite element modeling of creep behavior of FRP-externally strengthened reinforced concrete beams. Eng. Struct..

[4] Lin C, Li T, Chen S (2020). Structural identification in long-term deformation characteristic of dam foundation using meta-heuristic optimization techniques. Adv. Eng. Softw..

[5] Katouzian M, Vlase S, Scutaru ML (2021). Finite element method-based simulation creep behavior of viscoelastic carbon-fiber composite. Polymers.

[6] Rafiee R, Mazhari B (2016). Simulation of the long-term hydrostatic tests on glass fiber reinforced plastic pipes. Compos. Struct..

[7] Berardi VP, Perrella M, Feo L, Cricrì G (2017). Creep behavior of GFRP laminates and their phases: Experimental investigation and analytical modeling. Compos. Part. B-Eng..

[8] Jia Y, Peng K, Gong X, Zhang Z (2011). Creep and recovery of polypropylene/carbon nanotube composites. Int. J. Plast..

[9] Asyraf MRM, Ishak MR, Sapuan SM, Yidris N (2021). Comparison of static and long-term creep behaviors between balau wood and glass fiber reinforced polymer composite for cross-arm application. Fiber. Polym..

[10] Zhang YY, Sun Z, Li YQ, Huang P, Chen Q, Fu SY (2021). Tensile creep behavior of short-carbon-fiber reinforced polyetherimide composites. Compos. Part. B-Eng..

[11] Yang Z, Wang H, Ma X (2018). Flexural creep tests and long-term mechanical behavior of fiber-reinforced polymeric composite tubes. Compos. Struct..

[12] Harries KA, Guo Q, Cardoso D (2017). Creep and creep buckling of pultruded glass-reinforced polymer members. Compos. Struct..

[13] Ghosh SK, Rajesh P, Srikavya B, Rathore DK, Prusty RK, Ray BC (2018). Creep behavior prediction of multi-layer graphene embedded glass fiber/epoxy composites using time-temperature superposition principle. Compos. Part. A-Appl. S..

[14] Yu L, Ma Y (2019). Loading rate and temperature dependence of flexural behavior in injection-molded glass fiber reinforced polypropylene composites. Compos. Part. B-Eng..

[15] Asyraf MRM, Ishak MR, Sapuan SM, Yidris N (2021). Utilization of bracing arms as additional reinforcement in pultruded glass fiber-reinforced polymer composite cross-arms: creep experimental and numerical analyses. Polymers.

[16] Ferreira C (2001). Gene expression programming: A new adaptive algorithm for solving problems. Complex. Syst..

[17] Peng Y, Yuan C, Qin X, Huang J, Shi Y (2014). An improved gene expression programming approach for symbolic regression problems. Neurocomputing.

[18] Murad YZ (2021). Predictive model for bidirectional shear strength of reinforced concrete columns subjected to biaxial cyclic loading. Eng. Struct..

[19] Murad YZ (2020). Joint shear strength models for exterior RC beam-column connections exposed to biaxial and uniaxial cyclic loading. J. Build. Eng..

[20] Murad YZ, Tarawneh A, Arar F (2021). Flexural strength prediction for concrete beams reinforced with FRP bars using gene expression programming. Structures.

[21] Babanajad SK, Gandomi AH, Alavi AH (2017). New prediction models for concrete ultimate strength under true-triaxial stress states: An evolutionary approach. Adv. Eng. Softw..

[22] Iqbal MF, Liu QF, Azim I, Zhu X, Yang J, Javed MF, Rauf M (2020). Prediction of mechanical properties of green concrete incorporating waste foundry sand based on gene expression programming. J. Hazard. Mater..

[23] Wei Y, Xue X (2021). Permeability prediction in tight carbonate rocks using gene expression programming (GEP). Rock. Mech. Rock. Eng..

[24] Hassani M, Safi M, Ardakani RR, Daryan AS (2020). Predicting fire resistance of SRC columns through gene expression programming. J. Struct. Fire. Eng..

[25] Shahmansouri AA, Bengar HA, Ghanbari S (2020). Compressive strength prediction of eco-efficient GGBS-based geopolymer concrete using GEP method. J. Build. Eng..

[26] Mousavi SM, Aminian P, Gandomi AH, Alavi AH, Bolandi H (2012). A new predictive model for compressive strength of HPC using gene expression programming. Adv. Eng. Softw..

[27] Mansouri I, Güneyisi EM, Mosalam KM (2021). Improved shear strength model for exterior reinforced concrete beam-column joints using gene expression programming. Eng. Struct..

[28] Beheshti Aval SB, Ketabdari H, Asil Gharebaghi S (2017). Estimating shear strength of short rectangular reinforced concrete columns using nonlinear regression and gene expression programming. Structure.

[29] Tarawneh A, Almasabha G, Alawadi R, Tarawneh M (2021). Innovative and reliable model for shear strength of steel fibers reinforced concrete beams. Structure.

[30] Kara IF (2011). Prediction of shear strength of FRP-reinforced concrete beams without stirrups based on genetic programming. Adv. Eng. Softw..

[31] Yeddula BSR, Karthiyaini S (2020). Experimental investigations and GEP modelling of compressive strength of ferrosialate based geopolymer mortars. Constr. Build. Mater..

[32] Yeddula BSR, Karthiyaini S (2020). Experimental investigations and prediction of thermal behavior of ferrosialate-based geopolymer mortars. Arab. J. Sci. Eng..

[33] Güneyisi EM, Nour AI (2019). Axial compression capacity of circular CFST columns transversely strengthened by FRP. Eng. Struct..

[34] Nour AI, Güneyisi EM (2019). Prediction model on compressive strength of recycled aggregate concrete filled steel tube columns. Compos. Part. B-Eng..

[35] Jafari S, Mahini SS (2017). Lightweight concrete design using gene expression programing. Constr. Build. Mater..

[36] Gholampour A, Gandomi AH, Ozbakkaloglu T (2017). New formulations for mechanical properties of recycled aggregate concrete using gene expression programming. Constr. Build. Mater..

[37] Tan H, He L, Huang ZC, Zhan H (2021). Online signature verification based on dynamic features from gene expression programming. Multimed. Tools. Appl..

[38] Du X, Ni Y, Xie D, Yao X, Ye P, Xiao R (2015). The time complexity analysis of a class of gene expression programming. Soft. Comput..

[39] Anand A, Banerjee P, Sahoo D, Rathore DK, Prusty RK, Ray BC (2019). Effects of temperature and load on the creep performance of CNT reinforced laminated glass fiber/epoxy composites. Int. J. Mech. Sci..

[40] Berardi VP, Perrella M, Armentani E, Cricrì G (2021). Experimental investigation and numerical modeling of creep response of glass fiber reinforced polymer composites. Fatigue. Fract. Eng. M..

[41] Yao YF, Cheng H, Lin J, Ji JC (2021). Optimization of Burgers creep damage model of frozen silty clay based on fuzzy random particle swarm algorithm. Sci. Rep..

[42] Li G, Wang Y, Wang D (2021). Creep damage model of rock with varying-parameter under the step loading and unloading conditions. Sci. Rep..

[43] Biswas S, Fernandez Castellanos D, Zaiser M (2020). Prediction of creep failure time using machine learning. Sci. Rep..

